# Spatiotemporal clustering and correlates of childhood stunting in Ghana: Analysis of the fixed and nonlinear associative effects of socio-demographic and socio-ecological factors

**DOI:** 10.1371/journal.pone.0263726

**Published:** 2022-02-08

**Authors:** Fiifi Amoako Johnson

**Affiliations:** Department of Population and Health, Faculty of Social Sciences, College of Humanities and Legal Studies, University of Cape Coast, Cape Coast, Ghana; University of Colorado at Denver, UNITED STATES

## Abstract

Childhood stunting remains a major public health issue in many low- and middle-income countries. In Ghana, the progress made is insufficient to reach the targets set by the 2025 World Health Assembly and the 2030 United Nations Sustainable Development Goals. Although studies have examined the socio-demographic determinants of childhood stunting, there has not been any systematic study to examine the spatial associative effects of the socio-demographic and socio-ecological factors at the district level, where health programmes are implemented and monitored. Bayesian geo-additive semiparametric regression technique was used to analyse five conservative rounds of Demographic and Health Surveys in Ghana, with socio-ecological covariates derived from the Demographic and Health Survey Program Geospatial Covariate datasets to examine the temporal trends in childhood stunting, the extent of geospatial clustering at the district level and their associative relationships with socio-demographic and socio-ecological factors. The findings show that childhood stunting in Ghana is not spatially randomly distributed but clustered. Clustering of high childhood stunting was observed amongst districts in the Upper West, Upper East, Northern, North East, Savannah, and Western North regions, whilst clustering of low childhood stunting was observed in districts in the Greater Accra, Volta, Bono and the Eastern regions. Whist socio-demographic factors were predominantly associated with clustering of districts with high childhood stunting, the socio-ecological factors were mainly associated with clustering of districts with low childhood stunting. The socio-ecological factors identified to have a nonlinear associative effect with childhood stunting were Insecticide Treated Net (ITN) coverage, nightlight composite, travel time to a main settlement and population density. The findings suggest that targeted interventions at the district level are essential to reducing childhood stunting in Ghana.

## Introduction

This study examined the spatiotemporal clustering and socio-demographic and socio-ecological factors associated with district-level spatial patterns of childhood stunting in Ghana. Childhood growth faltering, often measured by stunting (low height-for-age) and wasting (low weight-for-height), is a reflection of social inequalities and a major public health challenge in many low- and middle-income countries [1]. Globally, estimates show that 144 million children (21.3%) under the age of five years were stunted in 2019, whiles 47 million were wasted in the same year [2]. Although the number of children with faltered growth has declined since 2000 (199.5 million children were estimated to be stunted in 2000), the progress made is insufficient to reach the targets set by the 2025 World Health Assembly (WHA) and the 2030 United Nations (UN) Sustainable Development Goals (SDGs) [2]. The global nutrition target set by the 2025 WHA and 2030 UN SDG is to achieve by 2025 a 40% reduction in the number of children under-five who are stunted [2].

Africa and Asia remain the most affected, with 54% and 40% of children estimated to be stunted, whilst 69% and 27% are wasted, respectively [2]. Research shows that stunted growth is a reflection of suboptimal health and nutritional disorder, associated with poor socioeconomic conditions, inadequate child feeding practices, poor maternal nutrition, and health, as well as increased risk of frequent and early exposure to illness [3]. On the other hand, wasting reflects recent severe weight loss, often associated with acute starvation or severe disease, but may also be caused by similar factors as stunting [3, 4]. Global and regional estimates confirm that stunting remains the most prevalent form of childhood malnutrition, whilst wasting is generally low even in developing countries, except in situations of severe food shortage [1, 2, 4].

Although there have been studies that examine the socio-demographic determinants of childhood stunting in Ghana, there has not been any systematic study to examine the spatial associative effects of the socio-demographic as well as the socio-ecological factors at the district level, where health interventions are implemented and monitored [5, 6]. Also, the nonlinear effects, which bring better insight into the associative relationships, have not been investigated [7]. Identifying the spatial effects of the socio-demographic and socio-ecological factors is essential for area-specific targeted interventions to strengthen childhood nutrition initiatives. This is particularly important as stunting is linked with other global and regional nutrition targets, including anaemia in women of reproductive age, low birth weight, childhood overweight, exclusive breastfeeding, and wasting [4].

The 2014 Ghana Demographic and Health Survey, using the new WHO Child Growth Standards estimates [8], reported a decline in stunting amongst children under five years of age, from 35% in 2003 to 19% in 2014. This is an indication that despite the decline, stunting continues to affect the lives of many children in the country [9]. Given the high level of childhood stunting in Ghana, both small- and large-scale studies have examined its socio-demographic determinants [10–15]. These studies show that economic and environmental factors including economic inequalities at the household level, maternal educational and occupational status, household hygiene, rural-urban residence, and access to health care services, are associated with childhood stunting in Ghana [10–15]. Additionally, demographic factors such as maternal and child age, sex, birth interval, parity, marital status, and the number of household members, among others, are also associated with childhood stunting [11, 16–18]. Nonetheless, the associative effects of the socio-ecological covariates which operate at the community level have not been systematically studied.

To effectively address the problem of childhood stunting in Ghana, there is the need to identify hot and cold spots of childhood stunting at the district level, where health intervention programmes are designed, implemented, and monitored. It is also essential that the associative effects of the socio-ecological factors, together with the socio-demographic factors, are examined. Analysing the fixed and nonlinear associative effects of the covariates is essential for understanding their underlining relationships with childhood stunting.

## Methods

### Data

The data for the analysis comes from five consecutive rounds of the Ghana Demographic and Health Survey (GDHS) and the Demographic and Health Survey (DHS) Program Geospatial Covariate datasets conducted in 1993, 1998, 2003, 2008, and 2014 [19, 20]. The socio-demographic covariates for the study were derived from the GDHS, whist the socio-ecological covariates were derived from the DHS Program Geospatial Covariate datasets. Demographic and Health Surveys (DHS) are nationally-representative household surveys that collect, analyse and disseminate data on a wide range of population and health issues in more than 90 countries. DHS undergoes comprehensive data quality checks (sampling, questionnaire design, survey procedures and methodological approaches) at the design, fieldwork, data processing and analysis stages to ensure robustness and national representativeness [21]. The GDHSs were implemented by the Ghana Statistical Service and Ghana Health Service (GHS), with technical assistance from the ICF International through The DHS Program [22].

The GDHS adopted a two-stage sampling approach using a systematic sampling procedure. At the first sampling stage, census enumeration areas, referred to as Primary Sampling Units (PSUs) or survey clusters, were selected. Households were then sampled from the selected clusters at the second stage of sampling [9]. The GDHS are nationally representative cross-sectional surveys that collect demographic and health information on women, men, children, and other household members [9]. The 1998, 2003, 2008, and 2014 GDHS collected information on anthropometric measures for all births five years preceding each survey, whilst the 1993 survey covered all births three years preceding the survey. The analysis covered 12,636 children (1993 GDHS = 1818, 1998 GDHS = 2626, 2003 GDHS = 3094, 2008 GDHS = 2385 and 2014 GDHS = 2713) for whom complete data were available. The DHS Program Geospatial Covariate datasets provide geospatial socio-ecological data for buffers surrounding 2 km of urban and 10 km of rural DHS survey clusters for the survey year [20]. These are standardised datasets that can be merged with DHS datasets at the community (cluster) level.

To ensure comparability across the selected surveys and to avoid distortion to the trends, the NCHS/CDC/WHO international reference standard (GDHS variable HW5) for assessing the nutritional status of children was adopted instead of the new WHO Child Growth Standards (GDHS variable HW70), which was applied only to the 2008 and 2014 GDHS [8]. The response variable was binary coded 1 (stunted) if a child’s height-for-age z-score was below minus two standard deviations of the World Health Organisation (WHO) child growth standards median and 0 otherwise [23]. The socio-demographic covariates for the analysis were selected based on literature evidence [10–18, 24, 25] and their availability in the GDHS datasets, whist the socio-ecological factors were selected based on the WHO Conceptual Framework for analysing stunting [26]. The selected covariates, their classification, and coding are shown in Tables 1 and 2.

**Table 1:** Socio-demographic covariate selected for the analysis.

Variable	Coding	Type of variable
Age of child in months		Continuous
Sex of child	0 = Male, 1 = Female	Categorical
Maternal age		Continuous
Had diarrhea recently	0 = No, 1 = Yes	Categorical
Had fever recently	0 = No, 1 = Yes	Categorical
Had cough recently	0 = No, 1 = Yes	Categorical
Age at first birth		Continuous
Age at first marriage		Continuous
Age at first sex		Continuous
Educational background	0 = No education, 1 = Primary, 2 = Secondary or higher	Categorical
Access to media information	0 = No access, 1 = Has access	Categorical
Religious affiliation	0 = No Religion, 1 = Catholic, 2 = Protestant, 3 = Other Christians, 4 = Islam, 5 = Traditionalist/Spiritualist	Categorical
Ethnicity	0 = Akan, 1 = Ga-Dangbe, 2 = Ewe-Guan, 3 = Mole-Dagbane, 4 = Grussi-Gruma-Hausa, 5 = Other	Categorical
Births in last five years	0 = One, 1 = Two, 2 = Three or more	Categorical
Antenatal visits for pregnancy	0 = No antenatal visit, 1 = 1–3 visits, 2 = 4–6 visits, 3 = More than 6 visits	Categorical
Place of delivery	0 = Home, 1 = Government facility, 2 = Private facility	Categorical
Paternal education	0 = No formal education, 1 = Primary, 2 = Secondary, 3 = Higher	Categorical
Type of marriage	0 = Monogamous, 1 = Polygamous, 2 = Not in union	Categorical
Household wealth status	0 = Poorest, 1 = Poor, 2 = Middle, 3 = Rich, 4 = Richest	Categorical
Place of residence	0 = Urban, 1 = Rural	Categorical
Age of household head		Continuous
Children under five years of age in the household	0 = 1 child, 1 = 2–3 children, 2 = 4 or more children	Categorical
Number of household members		Continuous
Number of living children		Continuous

Source of data: Ghana Demographic and Health Survey.

**Table 2:** Socio-ecological covariate selected for the analysis.

Variable	Definition
Built population	An index ranging from 0 to 1, where 0 represents extremely rural and to 1 represents extremely urban.
Day land surface temperature	Mean annual daytime land surface temperature.
Enhanced vegetation index	Density and condition of the greenness of an area.
Global human footprint	An index ranging between 0 (low) to 100 (high) covering human population pressure (population density), human land use and infrastructure (built-up areas, nighttime lights, land use/land cover), and human access (coastlines, roads, railroads, navigable rivers).
Irrigation	The average proportion of the area equipped for irrigation at the time.
ITN coverage	The proportion of people who slept under an insecticide-treated net the night before they were surveyed.
Land surface temperature	The average annual land surface temperature.
Malaria prevalence	The average parasite rate of plasmodium falciparum (PfPR) in children between the ages of 2 and 10 years old.
Nightlights composite	The average nighttime luminosity of the area shows the differentiation of regions based on the density of population and the degree of electrification of dwellings, commercial and industrial premises, and infrastructure. The indicator is a proxy measure of urbanisation and development, with higher intensity of nightlight reflecting higher degree of urbanisation and development.
Night land surface temperature	The average nighttime land surface temperature.
Proximity to national borders	The geodesic distance to the nearest international borders.
Proximity to protected areas	The geodesic distance to the nearest protected area (e.g., national parks, national forests, and national seashores) is defined by the United Nations Environment World Conservation Monitoring Centre.
Proximity to water	The geodesic distance to either a lake or the coastline.
Rainfall	The average annual rainfall.
Slope	Roughness the terrain.
Travel times	The average time (minutes) required to reach a settlement of 50,000 or more people.
Population density	The number of persons per square kilometer.

Source of data: The DHS Program Geospatial Covariate Datasets.

The covariates were grouped into socio-demographic and socio-ecological factors to examine their associations with the observed spatial patterns of childhood stunting at the district level. Potential multicollinearity amongst the continuous covariates was investigated using the Pearson correlation coefficient. Correlations amongst the other variables were examined using the Interval-by-Interval Pearson’s R, Ordinal-by-Ordinal Spearman Correlation, and Nominal by Interval Eta [27]. The correlations were not high enough for potential multicollinearity. The district map used for this study covers the 216 districts created in June 2012 and adopted for the last (2014) GDHS considered for this analysis.

### Statistical analysis

The percentage distribution of stunted children by the year of DHS survey and the fixed socio-demographic covariate were examined through cross-tabulation, using Chi-squared test to examine statistically significant differences by the levels of the covariates. Analysis of Variance (ANOVA) was used to examine the mean distributions of the continuous socio-demographic and socio-ecological covariates aggregated by the year of the survey and the stunting status of the children. Finally, the Bayesian Geoadditive Semiparametric (BGS) regression technique was used to examine the extent to which the socio-demographic and socio-ecological factors were associated with the observed spatial patterns of childhood stunting [28]. The advantage of the BGS technique is that it allows for simultaneous estimation of nonlinear effects of the continuous covariates as well as fixed effects of the categorical and continuous covariates in addition to the unobserved spatial effects, both spatially structured and unstructured [28].

The outcome variable of interest *y*_*ij*_ was coded 1 if child *i* in district *j* is stunted and 0 otherwise. The outcome variable *y*_*ij*_ follows a binomial distribution with the expected probability *π*_*ij*_ of being stunted. The logistic model linking the probability *π*_*ij*_ of a child being stunted is of the form

$$y_{ij}|\eta_{ij}∼B(\pi_{ij})$$ 1

$$\pi_{ij}=P(y_{ij}=1|\eta_{ij})=\frac{exp(\eta_{ij})}{1+exp(\eta_{ij})}$$ 2

where *η*_*ij*_ is the predictor of interest. If we have a vector $x_{ij}^{\prime }={\left(x_{ij1},\ldots ,x_{ijk}\right)}^{\prime }$ of *k* continuous covariates and $\lambda_{ij}^{\prime }={\left(\lambda_{ij1},\ldots \lambda_{ijd}\right)}^{\prime }$ a vector of *d* categorical covariates, then the predictor *η*_*ij*_ can be specified as

$$\eta_{ij}=\alpha \lambda_{ij}^{\prime }+\beta x_{ij}^{\prime }$$ 3

where *α* is a vector of unknown regression coefficients for the categorical covariates, $\lambda_{ij}^{\prime }$, *β* is a vector of unknown regression coefficients for the continuous covariates $x_{ij}^{\prime }$.

To account for nonlinear effects of the continuous covariate and the spatial correlation of the proportion of children stunted, the BGS framework, which replaces the strictly linear predictors with flexible semiparametric predictors, was adopted. The model is thus specified as

$$\eta_{ij}=\alpha \lambda_{ij}^{\prime }+f_{k}x_{ijk}^{\prime }+f^{spat(S_{i})}$$ 4

where *f*_*k*_(*x*) are nonlinear smoothing function (an approximating function that reduces randomness and allows patterns of associations to be identified) of the continuous variable *x*_*ijk*_ and $f^{spat(S_{i})}$ accounts for unobserved spatial heterogeneity at district *j* (*j* = 1,…,S), some of which may be spatially structured (correlated) and others unstructured (uncorrelated). The spatially structured effects show the effect of location by assuming that geographically close areas are more similar than distant areas, whilst the unstructured spatial effect accounts for spatial randomness in the model. Eq 5 is thus specified as

$$\eta_{ij}=\alpha \lambda_{ij}^{\prime }+f_{k}x_{ijk}^{\prime }+f^{str(S_{i})}+f^{unstr(S_{i})}$$ 5

where *f*^*str*^ is the structured spatial effects and *f*^*unstr*^ is the unstructured spatial effects and $f^{spat(S_{i})}=f^{str}+f^{unstr}$. The spatially structured effects depict the extent of childhood clustering and the associative effects of unaccounted predictor covariates, which may be spatially clustered ‎or random. The smooth effects of continuous factors are modelled with P-spline priors, whilst the spatial effects are modelled using Markov random field priors (correlated random effect for the spatial location of observation). P-splines are efficient for modelling nonlinear smooth effects of continuous covariates for varying coefficient models [29]. The approach uses a piecewise continuous function composed of many polynomials to establish the relationships of continuous covariates with the outcome variable instead of fitting one polynomial for all the data.

The posterior mode (the expected value of the parameter estimate) of the structured spatial effects and their corresponding probabilities at ‎‎a 95% nominal level were used to examine spatial correlations of the outcome variable at the district level. The posterior probabilities at the 95% nominal level show districts where childhood stunting was statistically significantly high (high positive estimates of the posterior mode), significantly low (high negative estimates of the posterior mode), and where the effects were not significant (estimated posterior mode not significantly different from zero). The estimated posterior mode of the spatial effects characterises unexplained spatially correlated covariate information. Thus, districts, where the socio-demographic and the socio-ecological factors were spatially correlated with childhood stunting were identified using a sequential modelling approach.

To examine if significant geospatial clustering exists in childhood stunting at the district level, a null model (constant model) was initially fitted (Model 0). Model 1 accounted for only the spatial effects. Model 2 included the year of the survey, whilst Model 3 added the socio-demographic factors. The socio-ecological factors were then included in Model 4. Only covariates significant at *p < 0*.*05* were retained in the model.

The Akaike weight *w*_*r*_ is used as the model selection criteria since it accounts for the spatial correlation of the variables [30, 31]. The Akaike weight *w*_*r*_ is formulated as

$$w_{r}=\frac{exp\{-\frac{1}{2}\Delta_{j}(AIC)\}}{\sum_{r=1}^{R}exp\{-\frac{1}{2}\Delta_{r}(AIC)\}}$$ 6

where Δ_*j*_(AIC) is the difference between the AIC for each model and the model with the lowest AIC, and *r* is the number of fitted models. The Akaike weight ranges between 0 and 1, with the sum of all candidate models equal to 1, and analogous to the probability that model *R*_*r*_ is the best model given the available data and all candidate models [32]. Interpretation of the final results was based on the best-fitted model. The statistical software R was used for the analysis [33].

## Results

### Bivariate analysis

Table 3 shows the weighted percentage of stunted children aggregated by year of survey and the selected categorical socio-demographic covariates. Chi-squared test was used to examine statistically significant differences by the categories of the covariates and across the year of the survey. The results show a statistically significant decline in childhood stunting for the 2008 and 2014 GDHS compared to that of the 1993, 1998, and 2003 GDHS. Male children were significantly more likely to be stunted for the 1993, 1998, and 2003 GDHS, but the differences were not important for the 2008 and 2014 GDHS. For all the surveys, twin children were significantly more likely to be stunted when compared to singletons. Where the results show statistically significant differences, children who were born by Cesarean section were less likely to be stunted. Generally, recent illnesses such as diarrhoea, fever, and cough had no significant association with childhood stunting. Consistently, maternal education had a statistically significant association with childhood stunting, with a decline in the percentage of children stunted as maternal education increases. Across all the surveys, children whose mothers had no access to media information were significantly more likely to be stunted. With regards to religious affiliation, statistically, significant differences were found in the 1998 (protestants less likely to be stunted, traditionalist/spiritualist more likely to be stunted), 2003 (no religion and traditionalist/spiritualist more likely to be stunted) and 2014 (traditionalist/spiritualist more likely to be stunted) GDHS. Other than the 2008 GDHS, there was a significant difference in childhood stunting by ethnicity. The results further show that the higher the number of births in the last five years, the higher the likelihood of the child being stunted, and the effects were statistically significant for the 1998, 2003, 2008, and 2014 GDHS. Consistently across all the surveys, the higher the number of antenatal visits, the lower the likelihood of a child being stunted. Similarly, children born in public and private health facilities were significantly less likely to be stunted when compared to those born at home, and the effects were statistically significant for all the surveys. Save for the 1993 GDHS; children were significantly more likely to be stunted in households where the number of children under five years was high. Paternal education was significantly associated with childhood stunting for all the surveys. Those whose fathers had secondary or higher education were less likely to be stunted compared to their counterparts. Also, household wealth status and place of residence were consistently associated with childhood stunting, with those belonging to the wealthier households and urban areas being less likely to be stunted.

**Table 3:** Percentage distribution of stunted children by year of DHS survey and socio-demographic characteristics.

Socio-demographic factors	1993 GDHS		1998 GDHS		2003 GDHS		2008 GDHS		2014 GDHS		Pooled data	
	% [95% CI]	n	% [95% CI]	n	% [95% CI]	n	% [95% CI]	n	% [95% CI]	n	% [95% CI]	n
Overall	26.6 [24.5, 28.6]	1818	26.3 [24.6, 28.0]	2626	30.0 [28.4, 31.6]	3094	23.1 [21.4, 24.8]	2385	13.5 [12.2, 14.8]	2713	23.9 [23.1, 24.6]	12636
Sex of child			**		**						**	
Male	28.4 [25.5, 31.3]	918	28.7 [26.2, 31.2]	1288	32.7 [30.4, 35.0]	1567	23.7 [21.3, 26.1]	1202	14.1 [12.3, 15.9]	1405	25.4 [24.3, 26.5]	6380
Female	24.7 [21.9, 27.5]	900	24.0 [21.7, 26.3]	1338	27.3 [25.1, 29.5]	1527	22.6 [20.2, 25.0]	1183	12.8 [11.0, 14.6]	1308	22.3 [21.3, 23.3]	6256
Type of birth	**		**						**		**	
Singleton	25.7 [23.7, 27.7]	1750	25.6 [23.9, 27.3]	2524	30.0 [28.4, 31.6]	2987	22.9 [21.2, 24.6]	2293	12.6 [11.3, 13.9]	2588	23.4 [22.6, 24.2]	12142
Twin	50.0 [38.1, 61.9]	68	43.6 [34.0, 53.2]	102	29.0 [20.4, 37.6]	107	29.2 [19.9, 38.5]	92	32.5 [24.3, 40.7]	125	36.0 [31.8, 40.2]	494
Birth by cesarean section			**		*				*		**	
No	26.9 [24.8, 29.0]	1740	26.9 [25.2, 28.6]	2530	30.4 [28.8, 32.0]	2996	23.3 [21.5, 25.1]	2241	14.1 [12.7, 15.5]	2412	24.5 [23.7, 25.3]	11919
Yes	19.2 [10.5, 27.9]	78	13.3 [6.5, 20.1]	96	19.8 [11.9, 27.7]	98	20.9 [14.3, 27.5]	144	9.6 [6.3, 12.9]	301	14.7 [12.1, 17.3]	717
Had diarrhea recently											**	
No	26.2 [23.9, 28.5]	1443	25.5 [23.6, 27.4]	2120	29.5 [27.7, 31.3]	2581	22.4 [20.5, 24.3]	1882	13.7 [12.3, 15.1]	2381	23.3 [22.5, 24.1]	10407
Yes	28.0 [23.5, 32.5]	375	30.0 [26.0, 34.0]	506	32.5 [28.4, 36.6]	513	26.2 [22.4, 30.0]	503	11.9 [8.4, 15.4]	332	26.6 [24.8, 28.4]	2229
Had fever recently											**	
No	25.8 [23.4, 28.2]	1320	25.3 [23.3, 27.3]	1872	30.1 [28.3, 31.9]	2418	22.8 [20.9, 24.7]	1885	13.5 [12.1, 14.9]	2298	23.2 [22.4, 24.0]	9793
Yes	28.7 [24.7, 32.7]	498	29.2 [26.0, 32.4]	754	29.5 [26.1, 32.9]	676	24.6 [20.8, 28.4]	500	13.6 [10.3, 16.9]	415	26.2 [24.6, 27.8]	2843
Had cough recently					**							
No	27.6 [25.3, 29.9]	1395	26.3 [24.3, 28.3]	1925	32.0 [30.1, 33.9]	2356	23.0 [21.1, 24.9]	1847	14 [12.6, 15.4]	2335	24.3 [23.5, 25.1]	9858
Yes	23.2 [19.2, 27.2]	423	26.4 [23.1, 29.7]	701	23.7 [20.6, 26.8]	738	23.6 [20.0, 27.2]	538	10.8 [7.7, 13.9]	378	22.5 [20.9, 24.1]	2778
Educational background	**		**		**		**		**		**	
No education	30.7 [27.3, 34.1]	691	32.7 [30.0, 35.4]	1194	38.7 [36.2, 41.2]	1447	25.8 [23.0, 28.6]	914	20.9 [18.3, 23.5]	973	30.7 [29.4, 32.0]	5219
Primary	25.5 [22.8, 28.2]	1018	27.7 [23.7, 31.7]	470	24.4 [21.1, 27.7]	653	27.3 [23.6, 31.0]	555	15.9 [12.9, 18.9]	561	24.4 [22.9, 25.9]	3257
Secondary or higher	10.1 [4.4, 15.8]	109	20.1 [17.6, 22.6]	962	24.1 [21.4, 26.8]	994	18.8 [16.3, 21.3]	916	8.4 [6.8, 10.0]	1179	17.1 [16.0, 18.2]	4160
Access to media information	**		**		**		*		**		**	
No access	31.9 [28.5, 35.3]	724	30.4 [28.2, 32.6]	1670	41.8 [37.8, 45.8]	574	27.5 [23.3, 31.7]	427	23.5 [19.1, 27.9]	354	31.4 [29.9, 32.9]	3749
Has access	23.0 [20.5, 25.5]	1094	20.5 [17.9, 23.1]	956	27.8 [26.1, 29.5]	2520	22.3 [20.5, 24.1]	1958	12.3 [11.0, 13.6]	2359	21.1 [20.3, 21.9]	8887
Religious affiliation			**		**				**		**	
No Religion	30.1 [24.6, 35.6]	272	33.2 [27.3, 39.1]	248	44.1 [37.9, 50.3]	247	30.4 [22.2, 38.6]	122	21.0 [13.7, 28.3]	118	33.0 [30.1, 35.9]	1007
Catholic	25.0 [20.1, 29.9]	304	26.8 [22.5, 31.1]	411	30.0 [25.6, 34.4]	417	21.6 [17.2, 26.0]	335	13.3 [9.7, 16.9]	346	24.2 [22.2, 26.2]	1813
Protestant	24.9 [21.5, 28.3]	638	19.0 [14.9, 23.1]	360	27.3 [22.9, 31.7]	395	22.1 [17.0, 27.2]	250	11.2 [7.4, 15.0]	261	21.7 [19.8, 23.6]	1904
Other Christians	26.2 [21.2, 31.2]	298	25.5 [22.7, 28.3]	938	25.7 [23.3, 28.1]	1256	21.5 [19.0, 24.0]	1004	12.7 [10.9, 14.5]	1295	21.3 [20.1, 22.5]	4791
Islam	29.1 [23.0, 35.2]	213	26.1 [21.6, 30.6]	359	35.4 [31.6, 39.2]	606	25.0 [21.1, 28.9]	467	13.2 [10.4, 16.0]	576	25.4 [23.6, 27.2]	2221
Traditionalist/ Spiritualist	28.0 [18.9, 37.1]	93	36.8 [31.4, 42.2]	310	41.3 [34.0, 48.6]	173	29.0 [22.8, 35.2]	207	28.0 [19.9, 36.1]	117	33.4 [30.3, 36.5]	900
Ethnicity	*		**		**				**		**	
Akan	27.9 [25.0, 30.8]	888	25.6 [23.1, 28.1]	1163	28.1 [25.6, 30.6]	1216	23.6 [20.9, 26.3]	922	13.0 [11.0, 15.0]	1041	23.5 [22.4, 24.6]	5230
Ga-Dangbe	22.9 [15.7, 30.1]	131	19.5 [13.5, 25.5]	167	22.9 [17.2, 28.6]	212	20.5 [12.9, 28.1]	109	4.8 [0.8, 8.8]	108	18.4 [15.6, 21.2]	727
Ewe-Guan	20.5 [16.0, 25,0]	303	20.8 [16.5, 25.1]	342	26.0 [21.8, 30.2]	424	18.6 [14.6, 22.6]	363	11.1 [7.9, 14.3]	379	19.4 [17.6, 21.2]	1811
Mole-Dagbane	30.5 [25.2, 35.8]	295	29.7 [24.7, 34.7]	319	38.0 [34.5, 41.5]	753	26.3 [22.8, 29.8]	607	14.6 [12.0, 17.2]	709	27.5 [25.8, 29.2]	2683
Grussi-Gruma-Hausa	29.8 [22.3, 37.3]	141	39.4 [34.6, 44.2]	406	38.9 [33.3, 44.5]	295	22.9 [17.9, 27.9]	271	21.1 [17.1, 25.1]	400	30.5 [28.2, 32.8]	1513
Other	18.3 [8.5, 28.1]	60	28.8 [22.9, 34.7]	229	29.8 [23.4, 36.2]	194	22.6 [14.9, 30.3]	113	14.3 [6.4, 22.2]	76	25.0 [21.7, 28.3]	672
Births in last five years			*		**		**		**		**	
One	26.8 [23.8, 29.8]	854	24.1 [21.8, 26.4]	1287	27.1 [24.8, 29.4]	1483	20.1 [17.8, 22.4]	1141	10.5 [8.9, 12.1]	1359	21.5 [20.5, 22.5]	6124
Two	25.4 [22.4, 28.4]	831	27.8 [25.2, 30.4]	1152	32.7 [30.3, 35.1]	1411	26.4 [23.8, 29.0]	1063	14.7 [12.7, 16.7]	1150	25.6 [24.5, 26.7]	5607
Three or more	32.3 [24.4, 40.2]	133	32.1 [25.4, 38.8]	187	33.0 [26.5, 39.5]	200	23.8 [17.6, 30.0]	181	25.5 [19.5, 31.5]	204	29.3 [26.3, 32.3]	905
Antenatal visits for pregnancy	**		**		**		**		**		**	
No antenatal visit	37.5 [31.4, 43.6]	240	35.0 [30.0, 40,0]	348	39.7 [36.7, 42.7]	1049	31.4 [28.1, 34.7]	738	21.8 [18.8, 24.8]	749	32.5 [30.9, 34.1]	3124
1–3 visits	27.3 [23.3, 31.3]	473	29.8 [26.2, 33.4]	626	30.9 [26.8, 35.0]	497	21.8 [17.1, 26.5]	300	17.2 [12.1, 22.3]	211	27.2 [25.3, 29.1]	2107
4–6 visits	28.0 [24.6, 31.4]	672	26.7 [23.8, 29.6]	921	27.0 [24.1, 29.9]	901	22.5 [19.6, 25.4]	776	9.6 [7.6, 11.6]	863	22.6 [21.3, 23.9]	4133
More than 6 visits	17.6 [14.0, 21.2]	433	19.7 [16.8, 22.6]	731	19.2 [16.2, 22.2]	647	15.0 [12.1, 17.9]	571	9.4 [7.5, 11.3]	890	15.7 [14.5, 16.9]	3272
Place of delivery	**		**		**		**		**		**	
Home	30.7 [27.9, 33.5]	1026	30.8 [28.5, 33.1]	1556	35.9 [33.7, 38.1]	1807	27.0 [24.4, 29.6]	1092	21.8 [19, 24.6]	848	30.4 [29.3, 31.5]	6329
Government facility	22.0 [18.6, 25.4]	586	20.9 [18.1, 23.7]	806	22.9 [20.3, 25.5]	1024	20.0 [17.6, 22.4]	1096	10.8 [9.3, 12.3]	1700	18.0 [17.0, 19.0]	5212
Private facility	18.9 [13.6, 24.2]	206	21.0 [16.1, 25.9]	264	23.3 [18.2, 28.4]	263	22.0 [16.2, 27.8]	197	7.1 [3.2, 11.0]	165	19.0 [16.7, 21.3]	1095
Children under five years of age in the household			**		**		**		**		**	
One	26.7 [23.3, 30.1]	662	22.1 [19.6, 24.6]	1045	25.2 [22.7, 27.7]	1155	19.4 [16.8, 22.0]	912	10.4 [8.6, 12.2]	1126	20.3 [19.2, 21.4]	4900
Two–three	25.7 [23.1, 28.3]	1061	27.6 [25.3, 29.9]	1439	32.9 [30.7, 35.1]	1792	25.5 [23.2, 27.8]	1352	15.0 [13.2, 16.8]	1483	25.6 [24.6, 26.6]	7127
4 or more	34.7 [25.1, 44.3]	95	49.1 [40.9, 57.3]	142	35.7 [28.0, 43.4]	147	27.0 [19.1, 34.9]	121	30.6 [21.7, 39.5]	104	35.8 [32.0, 39.6]	609
Type of marriage					**						**	
Monogamous	25.7 [23.3, 28.1]	1229	25.3 [23.3, 27.3]	1796	27.3 [25.4, 29.2]	2166	22.5 [20.5, 24.5]	1711	12.7 [11.2, 14.2]	1916	22.5 [21.6, 23.4]	8818
Polygamous	26.6 [22.5, 30.7]	436	27.5 [23.9, 31.1]	593	38.8 [35.1, 42.5]	673	23.2 [19.3, 27.1]	452	17.2 [13.8, 20.6]	462	27.8 [26.1, 29.5]	2616
Not in union	33.3 [25.8, 40.8]	153	31.3 [25.4, 37.2]	237	33.0 [27.2, 38.8]	255	27.0 [21.2, 32.8]	222	13.9 [10.2, 17.6]	335	26.4 [23.9, 28.9]	1202
Paternal education	**		**		**		*		**		**	
No formal education	31.6 [27.5, 35.7]	497	35.4 [32.2, 38.6]	846	39.2 [36.4, 42.0]	1138	26.7 [23.5, 29.9]	713	20.7 [17.9, 23.5]	785	31.6 [30.2, 33.0]	3979
Primary	26.8 [23.8, 29.8]	854	27.3 [21.3, 33.3]	214	33.2 [27.5, 38.9]	261	28.9 [22.7, 35.1]	206	18.7 [14.2, 23.2]	293	26.8 [24.8, 28.8]	1828
Secondary	20.7 [15.4, 26.0]	222	23.1 [20.8, 25.4]	1261	24.6 [22.3, 26.9]	1291	21.2 [18.8, 23.6]	1103	11.9 [10.1, 13.7]	1207	20.3 [19.2, 21.4]	5084
Higher	16.2 [9.5, 22.9]	117	16.8 [10.7, 22.9]	142	18.7 [12.3, 25.1]	144	17.6 [11.9, 23.3]	170	4.0 [1.5, 6.5]	227	13.5 [11.1, 15.9]	800
Don’t know/missing	25.0 [17.5, 32.5]	128	27.6 [20.7, 34.5]	163	31.7 [26.0, 37.4]	260	23.9 [17.9, 29.9]	193	9.0 [5.0, 13.0]	201	23.5 [20.8, 26.2]	945
Household wealth status	**		**		**		**		**		**	
Poorest	32.0 [27.1, 36.9]	353	38.8 [34.5, 43.1]	496	41.4 [38.4, 44.4]	1031	28.3 [25.1, 31.5]	783	19.6 [17.0, 22.2]	886	31.8 [30.3, 33.3]	3549
Poor	31.9 [27.4, 36.4]	408	31.4 [27.4, 35.4]	529	32.2 [28.7, 35.7]	694	30.2 [26.3, 34.1]	532	20.8 [17.5, 24.1]	573	29.3 [27.6, 31.0]	2736
Middle	29.1 [23.9, 34.3]	292	30.3 [26.3, 34.3]	510	30.8 [27.0, 34.6]	556	23.3 [19.1, 27.5]	384	11.3 [8.6, 14.0]	516	24.8 [23.0, 26.6]	2258
Rich	27.4 [22.9, 31.9]	372	24.6 [21.0, 28.2]	543	22.6 [18.6, 26.6]	425	16.2 [12.6, 19.8]	403	8.9 [6.1, 11.7]	403	19.9 [18.2, 21.6]	2146
Richest	13.5 [10.1, 16.9]	393	14.6 [11.6, 17.6]	548	13.9 [10.5, 17.3]	388	11.9 [8.1, 15.7]	283	4.6 [2.4, 6.8]	335	11.8 [10.4, 13.2]	1947
Place of residence	**		**		**		**		**		**	
Urban	15.9 [12.8, 19.1]	521	14.9 [12.0, 17.8]	582	20.4 [17.6, 23.1]	833	16.1 [13.5, 18.6]	791	9.3 [7.5, 11.0]	1089	14.9 [13.8, 16.0]	3816
Rural	30.8 [28.3, 33.4]	1297	30.1 [28.1, 32.1]	2044	34.6 [32.7, 36.6]	2261	27.4 [25.2, 29.6]	1594	17.1 [15.2, 18.9]	1624	28.5 [27.6, 29.5]	8820

CI–Confidence Interval

**p < 0.01

*p < 0.05.

The bivariate analyses of the continuous socio-demographic factors are shown in Table 4. The table shows that stunted children’s mean age was lower than non-stunted children, and the effects were statistically significant across all the surveys. The results further show that maternal age was significantly lower for mothers whose children were stunted when compared to those whose children were not stunted, with statistically significant effects in the 1993, 2008, and 2014 GDHS. The age of household heads was significantly associated with childhood stunting for the 2003 GDHS. Other than the 2003 GDHS, age at first birth was significantly associated with childhood stunting, with mothers of stunted children having a lower mean age at first birth compared to those who were not stunted. The results from the 1998, 2008, and 2014 GDHS show that age at first marriage was significantly associated with childhood stunting, revealing that the mean at first marriage for mothers of stunted was lower compared to those who were not stunted. Likewise, the 1993, 2008, and 2014 GDHS show that the mean age at first sex was statistically significantly lower for mothers of stunted children. Furthermore, the results further show that the mean number of household members and the number of living children were significantly associated with childhood stunting. The mean number of household members and the number of living children in a household was higher for stunted children than children who were not stunted.

**Table 4:** Mean distributions of the continuous socio-demographic covariates by year of DHS survey and stunting status.

Socio-demographic factors and Stunting status	1993 GDHS Mean [95% CI]	1998 GDHS Mean [95% CI]	2003 GDHS Mean [95% CI]	2008 GDHS Mean [95% CI]	2014 GDHS Mean [95% CI]	Pooled data Mean [95% CI]
Age of child (months)	**	**	**	**	**	**
Stunted	22.4 [21.6, 23.1]	34.6 [33.5, 35.7]	32.2 [31.2, 33.2]	34.1 [32.9, 35.4]	32.2 [30.6, 33.7]	25.1 [24.7, 25.4]
Not stunted	14.8 [14.2, 15.3]	25.5 [24.7, 26.3]	27.0 [26.3, 27.8]	27.6 [26.8, 28.4]	27.4 [26.7, 28.1]	31.5 [31, 32.1]
Maternal age	**			*	*	**
Stunted	26.5 [25.9, 27.1]	27.2 [26.7, 27.7]	28.2 [27.7, 28.6]	27.1 [26.5, 27.7]	27.5 [26.7, 28.2]	28.1 [27.9, 28.2]
Not stunted	27.5 [27.1, 27.8]	27.6 [27.3, 27.9]	28.1 [27.8, 28.4]	27.9 [27.6, 28.3]	28.4 [28.2, 28.7]	27.5 [27.3, 27.8]
Age of household head			**			*
Stunted	37.0 [35.9, 38.1]	40.3 [39.2, 41.3]	42.4 [41.4, 43.4]	39.3 [38.3, 40.3]	40.2 [38.8, 41.5]	40.1 [39.8, 40.3]
Not stunted	38.0 [37.3, 38.7]	39.6 [39.0, 40.2]	40.8 [40.2, 41.3]	39.9 [39.3, 40.5]	39.4 [38.9, 39.9]	40.7 [40.2, 41.2]
Age at first birth	*	*		**	**	**
Stunted	19.4 [19.1, 19.6]	19.4 [19.2, 19.7]	19.9 [19.7, 20.1]	19.7 [19.4, 20.0]	19.7 [19.3, 20.1]	20.2 [20.1, 20.2]
Not stunted	19.8 [19.6, 20.0]	20.0 [19.8, 20.2]	20.1 [19.9, 20.2]	20.3 [20.1, 20.5]	20.8 [20.6, 21.0]	19.7 [19.6, 19.8]
Age at first marriage		*		*	**	**
Stunted	18.2 [17.9, 18.5]	18.2 [18.0, 18.5]	18.6 [18.4, 18.9]	18.7 [18.4, 19.1]	18.8 [18.3, 19.2]	18.9 [18.9, 19]
Not stunted	18.5 [18.3, 18.7]	18.7 [18.5, 18.9]	18.9 [18.7, 19.1]	19.1 [18.9, 19.3]	19.9 [19.7, 20.1]	18.5 [18.3, 18.6]
Age at first sex	*			*	**	**
Stunted	16.5 [16.3, 16.7]	16.9 [16.7, 17.2]	17.4 [17.2, 17.6]	17.5 [17.2, 17.8]	17.1 [16.8, 17.4]	17.5 [17.5, 17.6]
Not stunted	16.7 [16.6, 16.8]	17.2 [17.1, 17.3]	17.6 [17.4, 17.7]	18.0 [17.8, 18.1]	17.9 [17.8, 18.1]	17.2 [17.1, 17.3]
Number of household members		**	**	*	**	**
Stunted	5.6 [5.4, 5.9]	6.1 [5.8, 6.3]	6.3 [6.1, 6.5]	6.1 [5.8, 6.3]	6.3 [6.0, 6.6]	5.9 [5.8, 5.9]
Not stunted	5.6 [5.4, 5.7]	5.5 [5.4, 5.7]	5.9 [5.7, 6.0]	5.7 [5.6, 5.8]	5.6 [5.4, 5.7]	6.3 [6.1, 6.4]
Number of living children	*	**	**	**	**	**
Stunted	3.1 [2.9, 3.3]	3.6 [3.4, 3.8]	3.7 [3.6, 3.9]	3.5 [3.4, 3.7]	3.7 [3.5, 3.9]	3.3 [3.3, 3.4]
Not stunted	3.1 [3.0, 3.2]	3.3 [3.2, 3.4]	3.4 [3.3, 3.5]	3.2 [3.2, 3.3]	3.2 [3.1, 3.3]	3.6 [3.5, 3.6]

CI–Confidence Interval

**p < 0.01

* p < 0.05.

The bivariate analyses of the continuous socio-ecological factors are shown in Table 5. The analyses revealed that the built population, global human footprint, irrigation, malaria prevalence, nightlights composite, proximity to protected areas, and travel times were statistically significantly associated with childhood stunting across all the selected surveys. In addition, the results show that the mean estimate for built population, global human footprint, irrigation, and nightlights composite were significantly lower for stunted children when compared to non-stunted children. However, the mean malaria prevalence, proximity to protected areas, and travel times were significantly higher for stunted children when compared to non-stunted children.

**Table 5:** Mean distributions of the continuous socio-ecological covariates by year of DHS survey and stunting status.

Socio-ecological factors	1993 GDHS Mean [95% CI]	1998 GDHS Mean [95% CI]	2003 GDHS Mean [95% CI]	2008 GDHS Mean [95% CI]	2014 GDHS Mean [95% CI]	Pooled data Mean [95% CI]
Built population	**	**	**	**	**	**
Not stunted	0.176 [0.159, 0.193]	0.165 [0.151, 0.179]	0.169 [0.156, 0.182]	0.209 [0.194, 0.224]	0.215 [0.202, 0.228]	0.188 [0.182, 0.195]
Stunted	0.082 [0.063, 0.101]	0.077 [0.062, 0.093]	0.086 [0.072, 0.100]	0.115 [0.094, 0.136]	0.120 [0.095, 0.145]	0.093 [0.085, 0.101]
Day land surface temperature		**	**			
Not stunted	30.1 [29.9, 30.2]	30.3 [30.2, 30.5]	29.9 [29.8, 30.1]	30.6 [30.4, 30.7]	31.3 [31.2, 31.5]	30.5 [30.4, 30.7]
Stunted	29.8 [29.5, 30.1]	30.7 [30.5, 31.0]	30.3 [30.1, 30.5]	30.5 [30.2, 30.8]	31.7 [31.3, 32.0]	30.5 [30.4, 30.6]
Enhanced vegetation index	**			**		
Not stunted	3287.7 [3229, 3346.5]	3330.4 [3285.0, 3375.9]	3511.7 [3467.8, 3555.6]	3430.8 [3382.3, 3479.2]	3470.3 [3427.8, 3512.7]	3418.1 [3397.1. 3439.1]
Stunted	3459.8 [3372, 3547.6]	3304.3 [3236.5, 3372.1]	3475.4 [3416.8, 3534.0]	3593.6 [3507.6, 3679.6]	3495.4 [3398.1, 3592.6]	3456.0 [3422.1, 3489.9]
Global human footprint	**	**	**	**	**	**
Not stunted	41.6 [40.5, 42.6]	39.6 [38.7, 40.4]	38.9 [38.1, 39.7]	41.4 [40.5, 42.3]	40.7 [39.9, 41.4]	40.3 [39.9, 40.7]
Stunted	35.4 [34.2, 36.7]	34.0 [33.0, 35.0]	33.6 [32.8, 34.5]	35.9 [34.6, 37.2]	35.5 [34.1, 36.9]	34.6 [34.2, 35.1]
Irrigation	**	**	**	**	**	**
Not stunted	1205.5 [1199.5, 1211.5]	1191.8 [1186.6, 1197]	1188.1 [1183.5, 1192.7]	1196 [1190.8, 1201.3]	1188.0 [1183.3, 1192.7]	1192.8 [1190.5, 1195.1]
Stunted	1190.2 [1181.1, 1199.2]	1166.5 [1158.3, 1174.6]	1167.4 [1161.1, 1173.7]	1179.2 [1170, 1188.4]	1164.6 [1152.8, 1176.4]	1172.5 [1168.7, 1176.3]
ITN coverage			**	*	**	**
Not stunted	0.116 [0.112, 0.120]	0.115 [0.112, 0.119]	0.113 [0.111, 0.116]	0.172 [0.168, 0.176]	0.588 [0.582, 0.595]	0.241 [0.237, 0.246]
Stunted	0.120 [0.115, 0.126]	0.120 [0.115, 0.124]	0.120 [0.117, 0.124]	0.180 [0.174, 0.186]	0.618 [0.604, 0.632]	0.192 [0.186, 0.199]
Land surface temperature	*	**	**			
Not stunted	25.1 [25.0, 25.3]	25.3 [25.2, 25.4]	25.3 [25.3, 25.4]	25.9 [25.8, 26.0]	26.3 [26.2, 26.4]	25.6 [25.6, 25.7]
Stunted	24.9 [24.7, 25.1]	25.5 [25.4, 25.7]	25.6 [25.4, 25.7]	25.8 [25.6, 25.9]	26.5 [26.2, 26.7]	25.6 [25.5, 25.7]
Malaria prevalence	**	**	**	**	**	**
Not stunted	0.574 [0.565, 0.582]	0.594 [0.587, 0.600]	0.476 [0.468, 0.484]	0.502 [0.491, 0.513]	0.288 [0.283, 0.292]	0.472 [0.468, 0.477]
Stunted	0.615 [0.603, 0.628]	0.630 [0.621, 0.639]	0.526 [0.516, 0.537]	0.569 [0.552, 0.587]	0.327 [0.316, 0.337]	0.547 [0.541, 0.554]
Nightlights composite	*p-value = 0*.*000*	*p-value = 0*.*000*	*p-value = 0*.*000*	*p-value = 0*.*000*	*p-value = 0*.*000*	*p-value = 0*.*000*
Not stunted	3.5 [3.1, 3.9]	2.8 [2.5, 3.0]	2.5 [2.2, 2.7]	3.2 [2.9, 3.4]	2.7 [2.5, 2.9]	2.87 [2.75, 2.99]
Stunted	1.5 [1.1, 1.9]	1.2 [0.9, 1.5]	1.1 [0.9, 1.3]	1.6 [1.2, 1.9]	1.4 [1.1, 1.8]	1.32 [1.18, 1.45]
Night land surface temperature	**			**		**
Not stunted	20.2 [20.2, 20.3]	20.3 [20.2, 20.3]	20.7 [20.7, 20.8]	21.2 [21.2, 21.3]	21.3 [21.2, 21.3]	20.8 [20.8, 20.8]
Stunted	20.0 [19.9, 20.1]	20.3 [20.3, 20.4]	20.8 [20.7, 20.9]	21.0 [21.0, 21.1]	21.2 [21.1, 21.3]	20.7 [20.6, 20.7]
Proximity to national borders	*		**			**
Not stunted	58.2 [55.3, 61.1]	53.1 [50.7, 55.5]	58.2 [55.9, 60.5]	58.1 [55.6, 60.6]	52.8 [50.7, 54.9]	56.9 [55.8, 54.8]
Stunted	65.3 [60.6, 70.1]	54.6 [50.8, 58.4]	64.1 [60.7, 67.6]	59.4 [55.1, 63.7]	51.9 [47.2, 56.6]	61.6 [59.7, 57.9]
Proximity to protected areas	**	**	**	**	*	**
Not stunted	59.8 [58, 61.7]	60.0 [58.4, 61.6]	58.7 [57.2, 60.2]	57.8 [56.2, 59.4]	56.8 [55.4, 58.1]	59.2 [58.5, 57.8]
Stunted	55.1 [52.3, 57.9]	53.8 [51.8, 55.8]	54.6 [52.6, 56.6]	52.2 [49.7, 54.8]	52.8 [50.2, 55.5]	54.9 [53.9, 52.8]
Proximity to water	*	**		**		**
Not stunted	58.5 [55.1, 61.8]	70.8 [67.4, 74.2]	74.9 [71.8, 78]	71.2 [67.8, 74.5]	74.5 [71.5, 77.6]	72.4 [70.9, 69.5]
Stunted	65.9 [60.3, 71.5]	89.5 [83.6, 95.4]	80.1 [75.6, 84.7]	84.6 [78.7, 90.6]	76.5 [68.9, 84.0]	83.0 [80.5, 77.9]
Rainfall	*	*			*	**
Not stunted	1289.5 [1276.8, 1302.2]	1063.6 [1053.2, 1074.0]	1132.7 [1122.7, 1142.7]	1331.4 [1319.9, 1342.9]	1002.1 [995.8, 1008.4]	1147.4 [1142.3, 1152.5]
Stunted	1316.5 [1296.6, 1336.5]	1085.7 [1071.4, 1100.1]	1134.1 [1120.8, 1147.4]	1352.0 [1332.7, 1371.3]	1022.9 [1009.2, 1036.6]	1175.8 [1167.5, 1184.1]
Slope						
Not stunted	0.556 [0.521, 0.590]	0.579 [0.549, 0.609]	0.504 [0.481, 0.528]	0.450 [0.425, 0.475]	0.440 [0.42, 0.461]	0.500 [0.489, 0.512]
Stunted	0.533 [0.485, 0.581]	0.547 [0.507, 0.587]	0.479 [0.444, 0.515]	0.498 [0.450, 0.546]	0.403 [0.36, 0.446]	0.497 [0.478, 0.517]
Travel times	**	**	**	**	**	**
Not stunted	130.7 [125.0, 136.4]	144.0 [139.1, 148.9]	144.9 [140.1, 149.6]	140.8 [135.4, 146.2]	48.7 [46.5, 50.8]	118.4 [116.3, 120.6]
Stunted	150.2 [142.5, 157.9]	177.1 [168.2, 186]	179.8 [171.4, 188.2]	160.3 [148.9, 171.8]	58.3 [53.2, 63.5]	156.0 [151.7, 160.4]

CI–Confidence Interval

**p < 0.01

* p < 0.05.

### Geospatial analysis

The estimated posterior odds ratios of childhood stunting and their corresponding 95% credible intervals for the fixed covariates are shown in Table 6, along with their model summary statistics. A sequential model building technique was used to analyse the associative effects of the socio-demographic and socio-ecological factors on childhood stunting. Interpretation of the model coefficients was based on the final model (Model 4).

**Table 6:** Posterior odds of child stunting for the fixed effects, their corresponding 95% credible intervals, and model summary statistics.

	Model 1	Model 2	Model 3 POR [95% CI]	Model 4 POR [95% CI]
**Fixed effects of the socio-demographic factors**				
Sex of child				
Male			1.00	1.00
Female			0.82 [0.75, 0.90]**	0.82 [0.75, 0.90]**
Type of birth				
Singleton			1.00	1.00
Twin			2.25 [1.82, 2.78] **	2.28 [1.84, 2.82]**
Birth interval				
First child			1.00	1.00
8–24 months			1.27 [1.00, 1.61] *	1.28 [1.01, 1.63] *
25–48 months			1.57 [1.32, 1.86] **	1.56 [1.32, 1.86] **
More than 48 months			1.31 [1.16, 1.47] **	1.31 [1.16, 1.48] **
Educational background				
No education			1.00	1.00
Primary			0.91 [0.80, 1.04]	0.91 [0.80, 1.04]
Secondary or higher			0.77 [0.67, 0.89] **	0.77 [0.66, 0.89] **
Religious affiliation				
No Religion			1.00	1.00
Catholic			0.79 [0.65, 0.95] *	0.80 [0.66, 0.97] *
Protestant			0.78 [0.64, 0.95] *	0.79 [0.65, 0.96] *
Other Christians			0.80 [0.67, 0.95] *	0.80 [0.67, 0.95] *
Islam			0.82 [0.67, 1.00] *	0.84 [0.69, 1.02]
Traditionalist/Spiritualist			0.90 [0.73, 1.12]	0.90 [0.72, 1.12]
Ethnicity				
Akan			1.00	1.00
Ga-Dangbe			0.90 [0.70, 1.16]	0.88 [0.69, 1.13]
Ewe-Guan			0.78 [0.64, 0.94] **	0.78 [0.65, 0.95] *
Mole-Dagbane			0.76 [0.62, 0.93] **	0.76 [0.62, 0.93] **
Grussi-Gruma-Hausa			0.80 [0.65, 0.99] *	0.81 [0.66, 1.00]
Other			0.82 [0.65, 1.05]	0.81 [0.64, 1.04]
Antenatal visits for pregnancy				
No antenatal visit			1.00	1.00
Less than 4 visits			0.88 [0.77, 1.02]	0.88 [0.76, 1.02]
4 To 6 visits			0.83 [0.73, 0.94] **	0.82 [0.73, 0.93] **
More than 6 visits			0.70 [0.61, 0.81] **	0.71 [0.61, 0.82] **
Household wealth status				
Poorest			1.00	1.00
Poor			0.97 [0.85, 1.10]	0.97 [0.85, 1.11]
Middle			0.87 [0.75, 1.02]	0.88 [0.76, 1.03]
Rich			0.73 [0.61, 0.87] **	0.74 [0.62, 0.89] **
Richest			0.45 [0.36, 0.58]**	0.48 [0.38, 0.61] **
Sex of household head				
Male			1.00	1.00
Female			1.18 [1.06, 1.32] **	1.18 [1.06, 1.32] **
Place of residence				
Urban			1.00	1.00
Rural			1.28 [1.11, 1.48] **	1.27 [1.06, 1.50] **
	Model 1	Model 2	Model 3 POR [95% CI]	Model 4 POR [95% CI]
**Fixed effects of the contextual socio-ecological factors**				
Distance to nearest national border				
Less than 1 km				1.00
1–4.99 km				0.68 [0.50, 0.93] **
5–9.99 km				0.73 [0.53, 1.00]
10–20 km				0.74 [0.54, 1.01]
Greater than 20 km				0.79 [0.58, 1.08]
**Variance components**				
Structured nonlinear covariates				
** Socio-demographic factors**				
Year of survey		0.029	0.0274	0.01
Age of child in months			0.1954	0.20
Maternal age in years			0.0179	0.02
Birth order number			0.0081	0.01
** Socio-ecological factors**				
ITN coverage				0.02
Nightlights composite				0.01
Travel times				0.01
Population density				0.06
Structured Spatial Effects (SSE)	0.136	0.143	0.081	0.074
Unstructured Spatial Effects (USE)	0.093	0.073	0.042	0.032
% Change in SSE				
Model Summary Statistics				
Deviance	13,353.8	13,126.30	11,811.90	11778.00
AIC	13,563.3	13,330.00	12,053.60	12033.70
BIC	14,369.9	14,085.8	12,951.00	12938.50
GVC	1.094	1.075	0.970	0.970
Change in Deviance	395.2	227	1,314	33.90
Change in AIC	369.7	239	1,276	19.90
Change in BIC	NA	283	1,136	12.50
Akaike weight	0.00	0.00	0.00	1.00

Model 0: Deviance = 13931; AIC = 13933.

### Geospatial dependence in childhood stunting

**Fig 1:**
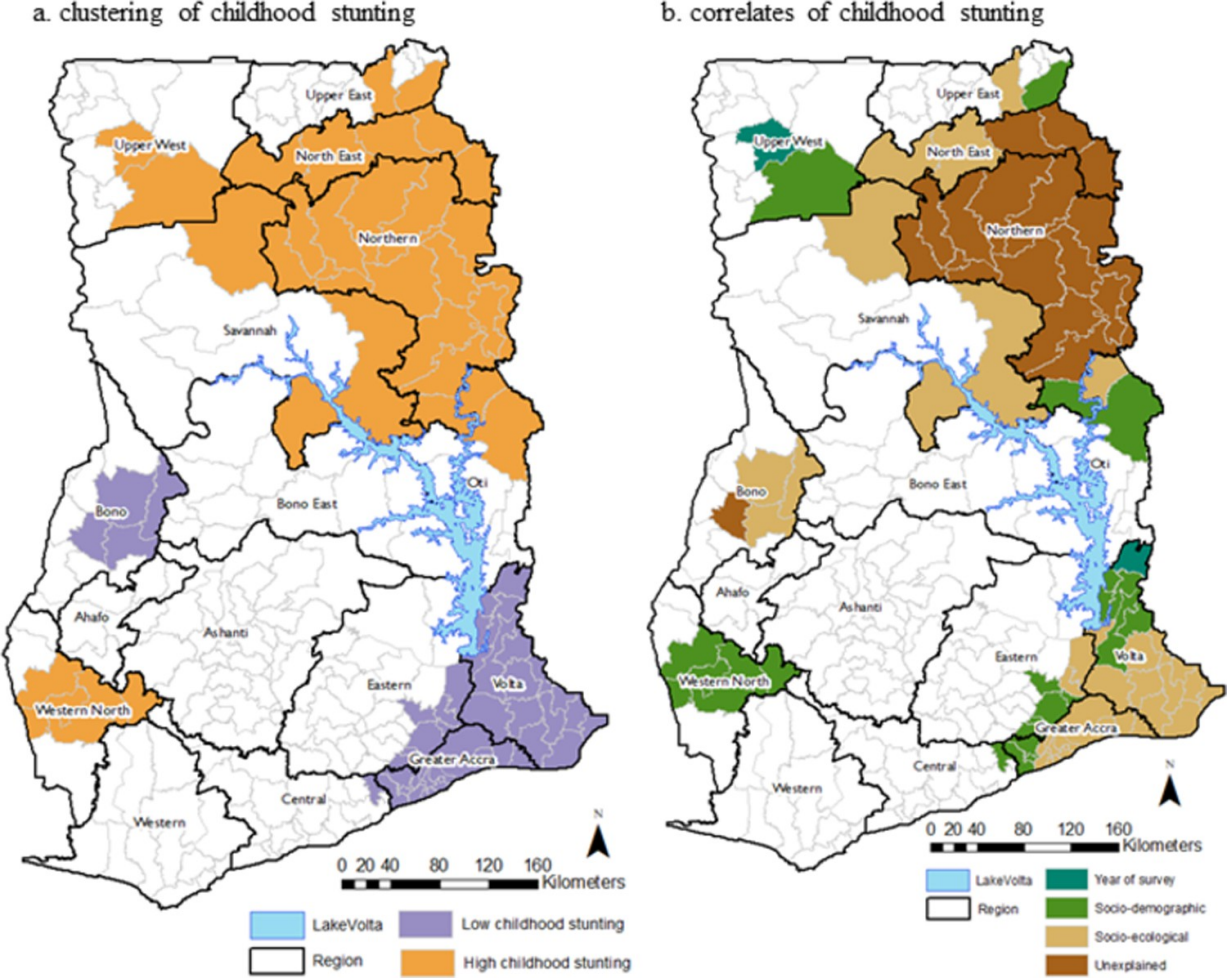
Geospatial (a) clustering of childhood stunting and (b) correlates of childhood stunting.

The estimated AIC for Model 0 (null model) was 13933.0 (Table 3). When the spatial effects were included in the model (Model 1), the AIC reduced by 369.7. The high reduction in the AIC after the spatial effects were included in the model, indicates that childhood stunting in Ghana is not spatially randomly distributed but clustered. Fig 1A shows districts where the posterior mode of the structured spatial effects were positive and significantly high (clusters of high childhood stunting) and negative and significantly low (clusters of low childhood stunting) at the 95% nominal level. The figure shows clustering of high childhood stunting within districts in the Upper West, Upper East, Northern, and Western North regions. Clustering of low childhood stunting was observed in districts in the Greater Accra, Volta, Bono, and those in the Eastern region bordering the Greater Accra and Volta regions.

### Temporal trends in childhood stunting

**Fig 2:**
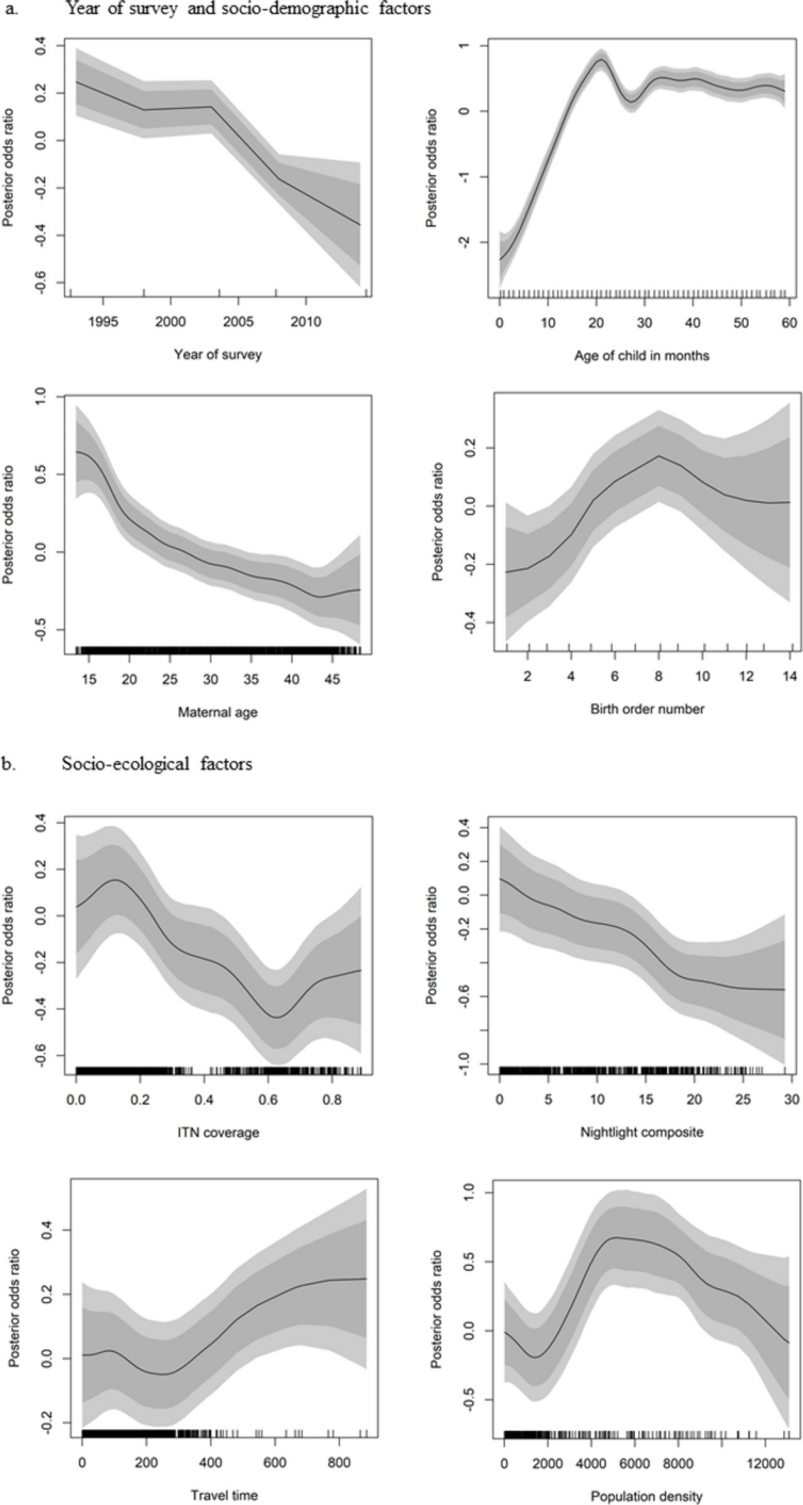
Posterior log odds of childhood stunting and their 95% and 80% credible interval for the non-linear covariates.

When the survey years were included in the model (Model 2), the AIC reduced by 239.0, indicating that the year of the survey has an important association with childhood stunting. Fig 2A shows a nonlinear temporal associative effect with childhood stunting. The figure depicts a declining trend in childhood stunting, with a significant decline after the 2003 GDHS. The posterior log-odds show that the odds of a child being stunted has declined from about 1.23 for the 1993 GDHS to about 0.82 for the 2014 GDHS, representing a decline in the odds by 34%. When the year of the survey was included in the model, the posterior mode of the structured spatial effects became statistically insignificant (p>0.05) in the Hohoe Municipal (low childhood stunting) and the Daffiama Bussie district (high childhood stunting) (Fig 1B).

### Associative effects of the socio-demographic factors with the observed spatial clustering of childhood stunting

The socio-demographic covariates were included in Model 3, leading to a reduction of 1,276.0 in the AIC. The fixed effects of the socio-demographic covariates that were statistically significant at *p<0*.*05* were the sex of the child, type of birth, birth interval, maternal educational attainment, religious affiliation, ethnicity, antenatal visits for pregnancy, household wealth status, sex of household head, and place of residence (Table 6). The important nonlinear covariates were the child’s age, maternal age, and birth order number (Table 6). The posterior odds ratio shows that female children had reduced odds of 0.18 of being stunted compared to males (Table 6, Model 4). Twin children were 2.28 times more likely to be stunted when compared to singletons. The results further revealed that, children with birth spacing of 8–24 months (POR = 1.28, 95% CI = 1.01, 1.63), 25–48 months (POR = 1.56, 95% CI = 1.32, 1.86) and more than 48 months (POR = 1.31, 95% CI = 1.16, 1.48) had higher odds of being stunted compared to firstborn children. Regarding the maternal factors, the results show that children whose mothers had secondary or higher education were 33% less likely to be stunted when compared to those whose mothers had no formal education. Concerning religion, the results show that children whose mothers were Catholics, Protestants, and Other Christians had reduced odds of 20%, 22%, and 20%, respectively, of being stunted compared to those whose mothers had no religious affiliation. Children whose mothers were of the Ewe-Guan and Mole-Dagbane ethnicity had lower odds of 21% and 20%, respectively, of being stunted compared to those whose mothers were Akan. High antenatal uptake was also associated with reduced odds of childhood stunting. Children whose mothers had four to six antenatal visits and those who had more than six visits had reduced odds of 18% and 29%, respectively, of being stunted compared to those who had no antenatal care. Considering the household factors, children who belong to rich households and households headed by men had lower odds of being stunted. Children who belong to the rich and richest households were 0.74 times and 0.48 times less likely to be stunted when compared to those from the poorest households. Children in households headed by females were 1.18 times more likely to be stunted when compared to those from households headed by males. On the place of residence, children from rural areas were 1.27 times more likely to be stunted when compared to their counterparts in urban areas.

The posterior mode of the structured spatial effects and their corresponding probabilities at 95% nominal level for Model 3, shows that, the socio-demographic covariates were spatially correlated with high childhood stunting in the Sefwi Akontombra, Sefwi-Wiawso, Sefwi Bibiani-Anhwiaso Bekwai, Juabeso, Suaman, and Bodi districts in the Western North Region, Nkwanta South district in the Oti region, Kpandai district in the Northern region, Garu Tempane in the Upper East region, and Wa East in the Upper West region (Fig 1B). In addition, the socio-demographic covariates are also spatially correlated with low childhood stunting in the Awutu Senya district in the Central region, Ga South, Ga West and Ga Central Municipal in the Greater Accra region, Ho Municipal, Ho West, Afadzato South, and North Dayi in the Volta region and Nsawam Adoagyiri, Akwapem North and Akwapem South district in the Eastern region.

### Associative effects of the socio-ecological factors with the observed spatial clustering of childhood stunting

When the socio-ecological covariates were added (Model 4), the AIC reduced by 19.90 (Table 6). All the significant socio-ecological covariates showed nonlinear associative effects, except for distance to the nearest national border. Distance to the nearest national border was included in the model as a categorical variable. The socio-ecological factors which exhibited nonlinear associative effects with childhood stunting were ITN coverage, nightlights composite, travel times, and population density (Table 6). The results show that children who reside more than 1 km away from a national border had reduced odds of being stunted. However, the effects were only significant for those between 1.00 to 4.99 km away from a national border (Table 6). Fig 2B shows a rise in the posterior log odds of a child being stunted where the proportion of people with an insecticide-treated net was between 0 and 0.1 (0–10%) and starts to decline with increasing coverage. The figure also shows a decline in the posterior log odds of a child being stunted with increasing nightlight composite. With regards to the average time (minutes) required to reach a settlement of 50,000 or more people (main settlement), the findings show that the posterior log odds of childhood stunting increases for those resident more than 220 minutes (4 hours) away from a main settlement. Regarding population density, the results show that the posterior log odds of childhood stunting decreased with population densities between 0 and 2000 persons per square kilometre but increased where the population density was between 2000 and 5000 persons per square kilometre and began to decline thereafter.

From Model 4, the posterior mode of the structured spatial effects and their corresponding probabilities at 95% nominal level show that the socio-ecological covariates were spatially correlated with high childhood stunting in the Nkwanta North district in the Oti region, the East Gonja, West Mamprusi, North Gonja, Mamprugu Moagduri districts in the Savannah region and Bawku West district in the Upper East region. The socio-ecological covariates were also spatially correlated with low childhood stunting in the Ga East, Accra Metropolis, Adenta, Ledzokuku/Krowor, Ashaiman, Tema Metropolis, Shai Osu Doku, Ada East, La Dade Kotopon, La Nkwantanang Madina, Kpone Katamanso, Ningo Prampram and Ada West districts in the Greater Accra region, the South Tongu, Keta Municipal, Ketu South, Ketu North, Akatsi South, Central Tongu, Agotime Ziope, South Dayi, North Tongu, Akatsi North and Adaklu districts in the Volta region and the Lower Manya and Asuogyaman districts in the Eastern region, the Sunyani West, Tain, and Wenchi districts in the Bono region.

### Observed spatial clustering of childhood stunting not explained by the socio-demographic and socio-ecological factors

The posterior mean of the structured spatial effects shows high childhood stunting in districts of Ghana’s Northern and North East regions, even after adjusting for the socio-demographic and socioeconomic covariates (Fig 1A and 1B). The districts in the Northern region with significantly high childhood stunting and not correlated with the covariates accounted for in the model were the Nanumba South, Nanumba North, Zabzugu, Yendi Municipal, Tamale North Sub Metro, Tolon, Savelugu Nanton, Karaga, Gushiegu, Saboba, Kumbumgu, Sagnerigu, Mion and Tatale districts. The Bunkpurugu Yonyo, Chereponi, and Mamprusi East districts in the North East region with significantly high childhood stunting and the Berekum district in the Bono region with significantly low childhood stunting were also not explained by the covariates accounted for in the model.

## Discussion

Previous studies have examined the socio-demographic factors associated with childhood stunting at the national level. However, in many low- and middle-income countries where health planning, implementation, and monitoring are decentralised [5, 6], there is the need to identify geographical clusters where childhood stunting is high and their associated factors. Also, socio-ecological factors are location-specific and potentially affect health outcomes at the local level, yet their associations with childhood stunting have not been systematically studied. Therefore, using five rounds of GDHS and DHS Program Geospatial Covariate datasets, this study has examined the spatiotemporal clustering of childhood stunting and the socio-demographic and socio-ecological factors associated with the observed spatial clustering.

The findings of the study show that childhood stunting in Ghana is not spatially randomly distributed but geographically clustered. Thirty-four districts were identified as clusters of high childhood stunting, whilst 46 were clusters of low childhood stunting. High childhood stunting was identified in districts in the northern part of Ghana (Upper West, Upper East, Northern, North East and the Savannah regions) and also the Western North region, whilst low childhood stunting was observed in districts in the Greater Accra, Volta, Bono, and the Eastern regions. The northern part of Ghana is characterised by high fertility, low access to health services, high infant, child and maternal mortality, high poverty levels, and low educational attainments, in contrast to the more developed southern part of the country [22, 34, 35]. In this regard, the clustering of high childhood stunting observed amongst districts in the northern part of the country concur with the demographic and socioeconomic characteristics of the region.

With regards to the temporal effects, the results show a declining trend in childhood stunting. Nonetheless, research suggests that the country remains off track to achieving national and international targets [36]. Evidence from this analysis indicates that the socio-demographic factors (sex of the child, type of birth, birth interval, maternal education, religious affiliation, ethnicity, antenatal visits for pregnancy, household wealth status, sex of household head, and place of residence) are associated with high childhood stunting in 10 of the 34 districts identified as clusters of high childhood stunting. In these districts, children of mothers with no formal education or primary education, no religious affiliation, no antenatal visits, or less than four antenatal visits, those from poor households and rural communities, are significantly more likely to be stunted. On the other hand, the socio-demographic factors are also associated with clustering low childhood stunting in 13 of the 46 districts with low childhood stunting. These findings concur with previous evidence that childhood malnutrition is predominantly associated with socioeconomic inequalities, particularly poverty and the lack of and use of health care services [13, 16, 36–38].

Regarding the socio-ecological factors, they were observed to be associated with childhood stunting in six of the 34 districts identified as clusters of high childhood stunting, and 29 of the clusters identified as clusters of low childhood stunting. The statistically significant socio-ecological factors (distance to nearest border, ITN coverage, nightlights composite, travel times, and population density) associated with childhood stunting mainly reflect urban development, industrialisation, and access to amenities [39]. [39] concluded that rapid urbanisation in Africa is not only changing its demographics but also its nutritional landscapes. For example, in Nigeria, using nightlight intensity (referred to in this study as nightlight composite), [39] reported a decline in childhood stunting with increasing light intensity, up to an intermediate level, but rising levels of childhood stunting at higher levels of light intensity. This indicates that, as cities grow much bigger, the benefits of urbanisation in reducing child stunting become less important. Contrary to the findings of [39], the findings of this study show that in Ghana, the benefit of urbanisation in reducing childhood stunting remains important as cities grow larger. The findings also show that the impact of ITN coverage on childhood stunting is less effective where less than 10% of the population use insecticide-treated nets. The results further show an increased odd of childhood stunting with increased distance to main settlements, indicating a lack of access to essential services and facilities [13, 38].

The findings from this study assert the need for targeted interventions to reduce child stunting in Ghana. This study, having identified districts where childhood stunting is strongly clustered and further revealed the socio-demographic and socio-ecological factors spatially associated with the observed clustering, will aid efforts to reduce childhood stunting, particularly targeting at-risk populations.

## Conclusions

This study systematically examined the temporal trends in childhood stunting, spatial clustering, and the spatial associative effects of the socio-demographic and socio-ecological factors at the district level, where health policies are implemented and monitored, using the Bayesian Geo-additive Semiparametric regression technique. The study shows that childhood stunting in Ghana is not spatially randomly distributed but clustered. The socio-demographic factors associated with the observed spatial patterns were predominantly associated with clustering of high childhood stunting, whist the socio-ecological factors were mainly associated with clustering of low childhood stunting. Targeted interventions to strengthen childhood nutrition programmes are essential, particularly amongst districts in the northern part of the country, where there is an observed strong clustering of districts with high childhood stunting. These districts should be targeted with both direct (nutritional education and provision of supplementary food) and indirect (malaria prevention, hygiene promotion and good quality water and sanitation) nutrition strategies. In addition, given the observed association between childhood stunting and low uptake of antenatal care, community outreach programmes targeting the poor and less educated women in rural communities may be essential.
